# Searching Method for Three-Dimensional Puncture Route to Support Computed Tomography-Guided Percutaneous Puncture

**DOI:** 10.3390/jimaging10100251

**Published:** 2024-10-14

**Authors:** Yusuke Gotoh, Aoi Takeda, Koji Masui, Koji Sakai, Manato Fujimoto

**Affiliations:** 1 Faculty of Environmental, Life, Natural Science and Technology, Okayama University, 3-1-1, Tsushima-naka, Kita-ku, Okayama 7008530, Japan; 2 Graduate School of Natural Science and Technology, Okayama University, 3-1-1, Tsushima-naka, Kita-ku, Okayama 7008530, Japan; takeda-a@s.okayama-u.ac.jp; 3 Department of Radiology, Kyoto Prefectural University of Medicine, Kajii-cho, Kawaramachi-Hirokoji, Kamigyo-ku, Kyoto 6028566, Japan; mc0515kj@koto.kpu-m.ac.jp (K.M.); sakai3@koto.kpu-m.ac.jp (K.S.); 4 Graduate School of Informatics, Osaka Metropolitan University, 1-1 Gakuen-cho, Nakaku, Sakai, Osaka 599-8531, Japan; manato@omu.ac.jp; 5 RIKEN Center for Advanced Intelligence Project AIP, 1-4-1 Nihon-bashi, Tokyo 103-0027, Japan

**Keywords:** CT-guided percutaneous puncture, searching method, three-dimensional puncture route

## Abstract

In CT-guided percutaneous punctures—an image-guided puncture method using CT images—physicians treat targets such as lung tumors, liver tumors, renal tumors, and intervertebral abscesses by inserting a puncture needle into the body from the exterior while viewing images. By recognizing two-dimensional CT images prior to a procedure, a physician determines the least invasive puncture route for the patient. Therefore, the candidate puncture route is limited to a two-dimensional region along the cross section of the human body. In this paper, we aim to construct a three-dimensional puncture space based on multiple two-dimensional CT images to search for a safer and shorter puncture route for a given patient. If all puncture routes starting from a target in the three-dimensional space were examined from all directions (the brute-force method), the processing time to derive the puncture route would be very long. We propose a more efficient method for three-dimensional puncture route selection in CT-guided percutaneous punctures. The proposed method extends the ray-tracing method, which quickly derives a line segment from a given start point to an end point on a two-dimensional plane, and applies it to three-dimensional space. During actual puncture route selection, a physician can use CT images to derive a three-dimensional puncture route that is safe for the patient and minimizes the puncture time. The main novelty is that we propose a method for deriving a three-dimensional puncture route within the allowed time in an actual puncture. The main goal is for physicians to select the puncture route they will use in the actual surgery from among the multiple three-dimensional puncture route candidates derived using the proposed method. The proposed method derives a three-dimensional puncture route within the allowed time in an actual puncture. Physicians can use the proposed method to derive a new puncture route, reducing the burden on patients and improving physician skills. In the evaluation results of a computer simulation, for a 3D CT image created by combining 170 two-dimensional CT images, the processing time for deriving the puncture route using the proposed method was approximately 59.4 s. The shortest length of the puncture route from the starting point to the target was between 20 mm and 22 mm. The search time for a three-dimensional human body consisting of 15 CT images was 4.77 s for the proposed method and 2599.0 s for a brute-force method. In a questionnaire, physicians who actually perform puncture treatments evaluated the candidate puncture routes derived by the proposed method. We confirmed that physicians could actually use these candidates as a puncture route.

## 1. Introduction

Image-guided puncture, in which ultrasonography (US) and computed tomography (CT) images are used as guides, is currently used in clinical practice. In a puncture treatment, a needle-like medical device called a puncture needle is inserted into the body from the exterior to biopsy cells to inject drugs and irradiate tumors. In this way, physicians can treat targets such as lung tumors, liver tumors, renal tumors, and intervertebral abscesses while viewing images. Image-guided puncture techniques allow physicians to visualize the interior of the body and accurately puncture lesions that cannot be seen from the exterior.

Although US images have been used for image-guided puncture, there is a limit to the areas of the body that can be displayed in US images. Recently, CT-guided percutaneous punctures [1], which is an image-guided puncture method using CT images, have become the main method used. Physicians can take CT images of any part of the body with almost no limitation. Therefore, CT-guided percutaneous punctures can theoretically be used to puncture targets located anywhere in the body. In the current CT-guided percutaneous puncture technique, the physician determines the least invasive puncture route by viewing two-dimensional CT images prior to the procedure. Consequently, the potential puncture route is limited to a two-dimensional area along a cross section of the human body.

We address the key problem of finding the optimal puncture route. In the shortest route problem with obstacle weights, an algorithm [2] was proposed to find the route with the shortest Manhattan distance between two points. Bose et al. [3] proposed a method for deriving the shortest path in a discretized continuous two-dimensional space divided into weighted triangles. Algorithms [4,5] were also proposed for determining the route of a robot and the locations of relay base stations in wireless communications. Lubiw et al. [6] proposed a method for drawing a planar straight line of the graph inside the polygonal region. However, these algorithms do not find the shortest route that avoids all obstacles but rather the route with the minimum number of turns, considering only horizontal or vertical line segments.

In this paper, we aim to search for a safe puncture route with a short puncture distance for the patient by constructing a three-dimensional puncture space. To search for a puncture route in a three-dimensional puncture space, such a space is constructed based on multiple two-dimensional CT images. This kind of search method adopts three-dimensional CT images that are converted from multiple two-dimensional CT images using volume rendering technology. These images are used to identify areas in the body that cannot be punctured, such as bones, blood vessels, and organs. The method also searches outward from the target as a starting point to derive a possible puncture route, and it derives a candidate route with a short distance from the starting point of the puncture. When searching for a three-dimensional puncture route, all routes starting from the target in three-dimensional space are examined by brute force. Therefore, the processing time required to derive a puncture route in the brute-force method is very long. Li et al. [7] used Graphics Processing Units (GPUs) to reduce the processing time needed to search for three-dimensional puncture routes.

The current problem is that there is no method to derive a three-dimensional puncture route within the time required for the physician to decide on the puncture route prior to surgery. As a background, in a three-dimensional puncture, the number of voxels that determine whether the puncture is possible or not on the route from the puncture start position to the target is much larger compared to a two-dimensional puncture. Therefore, the conventional method of searching for all possible routes may not be able to complete the search for the puncture route within the allowed time. The issue to be solved is how to derive a candidate for a three-dimensional puncture route within the allowed time. We needed an algorithm that efficiently searches for a puncture route while reducing the number of voxels searched.

The major gap in the current research corpus concerns studies that use three-dimensional CT images created by combining multiple two-dimensional CT images to explore puncture routes. There is a research gap because it can contribute to the construction of a new puncture technique that is safer and takes less time by deriving a puncture route that physicians have not thought of before.

Here, we propose a search method that applies a ray-tracing approach to find a three-dimensional puncture route in a CT-guided percutaneous puncture. Specifically, the proposed method leverages the key strength of ray tracing, i.e., to quickly derive a line segment from a given start point to an end point in a two-dimensional plane, by applying it to three-dimensional space.

The main novelty is that we proposed a method for deriving a three-dimensional puncture route within the allowed time in an actual puncture. The main goal is for physicians to select and puncture route they will use in the actual surgery from among the multiple three-dimensional puncture route candidates derived using the proposed method.

Our contributions in this paper are as follows:

- The proposed method allows a physician to derive a three-dimensional puncture route that is safe for the patient and minimizes the puncture time using all CT images of the human body within the actual search time for the puncture route;
- In a questionnaire-based evaluation, a physician experienced in puncture treatments responded that the candidate routes identified by the proposed method could actually be used as puncture routes.

The remainder of the paper is organized as follows. First, we explain the CT-guided percutaneous puncture method in Section 2. The conventional route-searching method for a three-dimensional puncture is described in Section 3. Next, related work is presented in Section 4. In Section 5, we introduce our proposed searching method for a three-dimensional puncture route in a CT-guided percutaneous puncture. We evaluate our proposed method in Section 6 and discuss its limitations and potential in Section 7. Finally, we offer our conclusions in Section 8.

## 2. CT-Guided Percutaneous Puncture

We explain an overview of the CT-guided percutaneous puncture method. The physician inserts the puncture needle and treats the target, such as a lung mass, liver mass, renal mass, or intervertebral abscess, while viewing the images. The needle is inserted from outside the skin into the mass or abscess indicated by the arrow. Image-guided puncture technology allows physicians to accurately puncture lesions that cannot be seen from outside the body while viewing images of the interior of the body. This technique is useful in medical practice for biopsies, abscess drainage, and the local treatment of cancer.

### Current Issues

There are two problems with the current methods of route derivation in CT-guided percutaneous punctures. The first one is that the area of possible punctures is limited. There are areas of the body that cannot be punctured, such as bones, blood vessels, and organs. Therefore, the puncture route from the starting point of the puncture to the target should be as parallel as possible to the CT cross section.

The puncturable or non-puncturable area is determined by the CT value [8], which is a value representing the density of the CT image. A two-dimensional CT image is composed of pixels, while a three-dimensional CT image is composed of voxels. These CT images are displayed in black and white based on the image density values. Accordingly, the CT values of body tissues such as bones and organs are expressed within a certain range.

**Figure 1:**
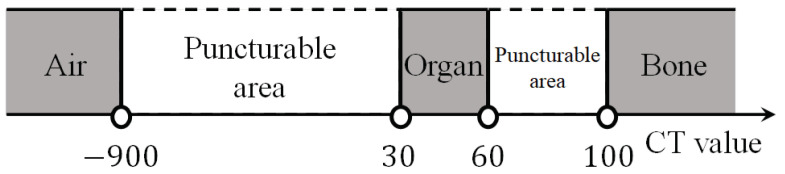
Puncturable areas based on CT values of body tissues.

The CT value set per voxel for each major type of tissue in the body is shown in Table 1. The CT value is set in the range of −2000 to 4000 and is displayed in black and white according to the size of the CT value. The range of the puncturable area based on the CT value for each body tissue affecting the puncture is shown in Figure 1. If the CT value is less than −900, the area is considered to be air and thus cannot be punctured. Moreover, the physician cannot puncture organs with CT values between 30 and 60 or bone areas with CT values greater than 100. Therefore, we set pixels with CT values between −900 and 30 and between 60 and 100 as puncturable areas.

The second problem with current methods is that physicians are unable to determine the reliability of the puncture route they have chosen. The puncture route varies greatly according to the experience of physicians. In fact, the puncture routes confirmed by several physicians are actually used. In this paper, we propose a searching method to help physicians determine the puncture route with confidence.

## 3. Methods of Searching for Routes in Three-Dimensional Puncture

Before the procedure, a physician uses two-dimensional CT images to determine the puncture route that will cause the least amount of stress to the patient. Therefore, the candidate puncture route is limited to a two-dimensional area along a cross section of the human body. In this paper, we aim to construct a three-dimensional puncture space based on multiple two-dimensional CT images to search for a safer and shorter puncture route for the patient.

Three-dimensional puncture route selection uses volume rendering technology to convert two-dimensional CT images into three-dimensional images. Such a three-dimensional CT image is used to identify areas in the body that cannot be punctured, such as bones, blood vessels, and organs. Next, the search method uses the target as a starting point and searches outward to derive the possible puncture route. It also derives the candidate puncture route with the shortest distance from the starting position to the target.

Many physicians perform two-dimensional punctures, where the puncture route is determined based on a single CT image. On the other hand, by deriving the route in a three-dimensional puncture space using multiple CT images, physicians can greatly increase the number of candidate puncture routes and derive a puncture route that shortens the puncture distance and reduces the burden on the patient. However, the routes for three-dimensional punctures up to now have been determined based on the physician’s experience, and there has been no mechanism to search for all candidate puncture routes starting from the target in a three-dimensional area. Therefore, in the case of a brute-force search, the processing time for deriving a puncture route is very long.

### 3.1. Assumed Environment

To search for the optimal three-dimensional puncture route, we present an assumed environment.

- The CT image consists of two-dimensional data with a height and width of 512 pixels, and three-dimensional data can be created by combining multiple two-dimensional CT images;
- The CT value data set for each voxel that makes up the three-dimensional CT image is stored in an array;
- The target position is set based on the physician’s opinion;
- The chosen puncture route is determined by the physician from several candidate puncture routes.

### 3.2. Puncture Route

#### 3.2.1. Shortest Route

**Figure 2:**
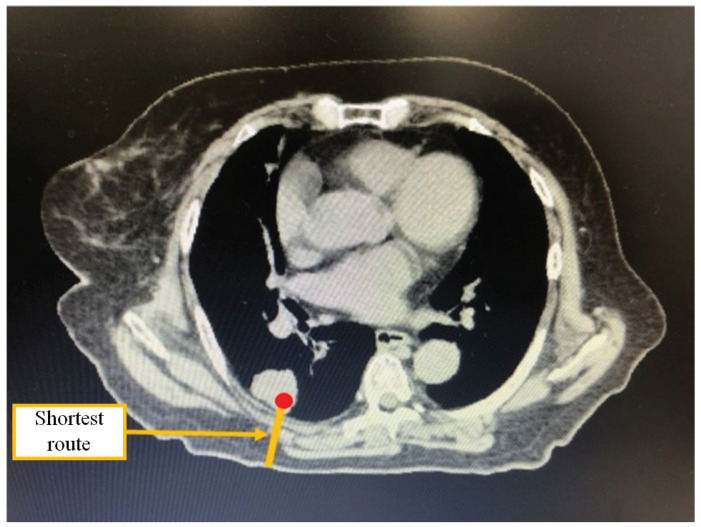
Example of displaying the shortest route.

An example of the shortest route in a CT image puncture is shown in Figure 2. The shortest route among multiple puncture routes from the pixel containing the coordinates of the target indicated by the red circle to the pixel in contact with the air outside the body is shown. By selecting the shortest route, the physician can shorten the puncture time and reduce the radiation dose from the CT scan during the actual puncture.

#### 3.2.2. Safe Route

**Figure 3:**
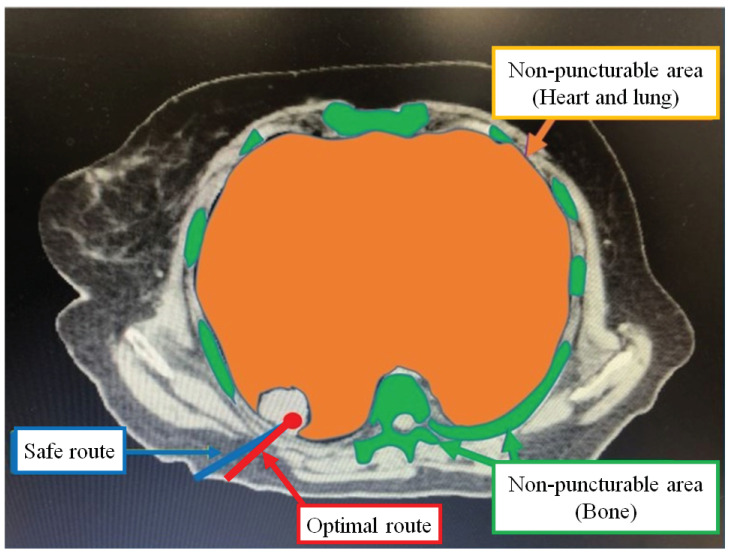
Example of a safe route and optimal route.

An example of a safe route for a CT image puncture is shown in Figure 3. The safe route has the longest distance away from a pixel on the puncture route to a pixel in the non-puncturable area. During the actual puncture, there is some deviation between the predefined puncture route and the trajectory of the puncture needle. The safe route naturally increases the puncture distance compared to the shortest route. On the other hand, since the possibility of puncturing a non-puncturable area is reduced, the physician can make the puncture more safely.

#### 3.2.3. Optimal Route

In Figure 3, we show an example of the optimal route for a CT image puncture. The optimal route is a route suitable for puncture according to many physicians based on the shortest route and the safe route. By selecting the optimal route, the physician can perform the puncture while giving consideration to both shortening the puncture distance and preventing medical accidents.

### 3.3. How to Search for Puncture Route in Brute-Force Method

**Figure 4:**
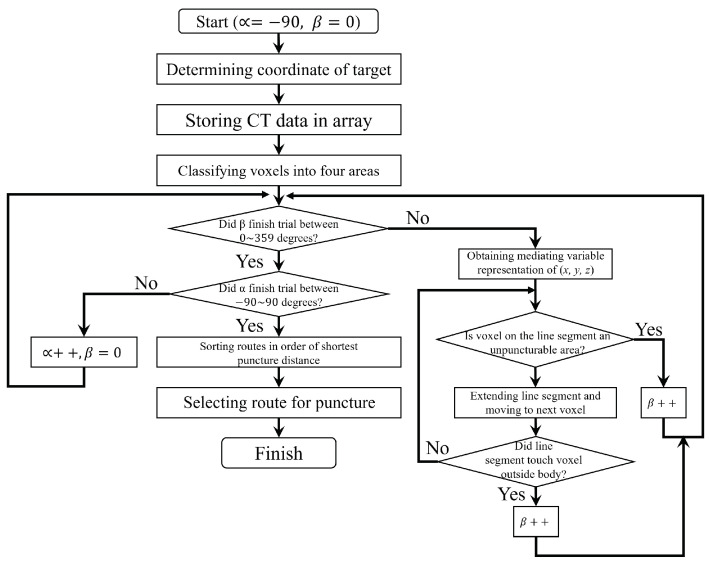
Flowchart of brute-force method.

The flowchart of the brute-force method is shown in Figure 4. The procedure for finding a three-dimensional puncture route in the brute-force method is as follows:

1. The coordinate of the target is determined based on the physician’s judgment;
2. The 4,439,040 (=512×512×170) CT data that compose the CT image containing the target are stored in the array;
3. Based on the values of the array data read in step (2), each voxel is classified into a puncturable area, a non-puncturable area, an area of air representing the exterior of the body, and a target area;
4. For a line segment of the puncture route set at a fixed length from the target, trials are repeated to rotate the mediating variable by one degree based on the mediating variable representation in the following equation. Let *R* be the radius of the sphere, α be an angle on the *z* axis, and β be an angle on the xy plane. In addition, let the range of the mediating variables be $-\frac{\pi }{2}\leq \alpha \leq \frac{\pi }{2}$ and 0≤β≤2.
  (1) $$\left\{\begin{array}{c} x=R\cos\alpha \cos\beta \\ y=R\cos\alpha \sin\beta \\ z=R\sin\beta \end{array}\right.$$
  In each trial, either (a) or (b) is performed based on the area containing each voxel tangent to the line segment.
  (a) If the voxel tangent to the segment is inside the non-puncturable region, the trial at the current angle is terminated and another trial is performed with the mediator variable rotated by 1 degree;
  (b) Otherwise, after extending the line segment from the target at the current angle, the trials are repeated from step 4.
5. If the line segment extended in step 4 touches a voxel outside the body, this line segment is used as a candidate puncture route and the length of the puncture route is calculated as the puncture distance;
6. Trials are performed for all angles centered on the target at 0∼359 degrees;
7. The routes are sorted in order of the shortest puncture distance from the target to the exterior of the body for the puncture routes that do not intersect the non-puncturable area;
8. The physician selects the route to be used for the puncture from the sorted routes.

## 4. Related Work

Kawata et al. [9] proposed a three-dimensional angiogram-processing algorithm using cone beam CT to reconstruct blood vessels in three dimensions from images measured from multiple directions as a processing technique. This algorithm can extract the direction of vessel movement, measure the vessel cross-sectional area, select the vessel route, and measure the distance of the route. In addition, this algorithm can support the quantitative diagnosis of vascular diseases and the needed treatment planning.

Zerobot [10] is a remote-controlled puncture robot for Interventional Radiology (IVR), in which a needle or catheter is inserted into the body using CT fluoroscopy or X-ray images. While robots help physicians to reduce radiation exposure during treatment, they cannot completely eliminate radiation exposure. The Zerobot can prevent radiation exposure to physicians by remotely controlling the entire process, from robot placement to needle insertion.

Hiraki et al. [11] developed a remote-controlled robot that considers the physician’s exposure to radiation as well as changes in the target’s position. The robot can adjust the needle position during insertion by guiding the puncture using real-time CT images.

The proposed method in this paper differs from conventional studies in that it searches for multiple three-dimensional puncture routes based on CT values in the body. In addition, by reducing the time required to search for the puncture route, physicians can treat more patients.

## 5. Proposed Method

In this research, to support CT-guided percutaneous punctures, we propose a searching method for a three-dimensional puncture route. The proposed method uses a ray-tracing method [12] to detect the voxel coordinates where a straight line passes through a three-dimensional area. Compared with the brute-force method, the proposed method reduces the time required to search for the puncture route and obtains the coordinates of the starting point of the puncture where the distance to the target is short.

First, the proposed method creates a three-dimensional CT image from multiple two-dimensional CT images by reconstruction and stereoscopic image display processing. Then, using the three-dimensional CT images, conditions are set to discriminate non-puncturable regions in the body, such as bones, blood vessels, and organs. For the puncturable regions, the proposed method derives multiple shortest routes, which are the routes with the shortest distance from the starting point of the puncture to the target.

### 5.1. Bresenham’s Algorithm

A Digital Differential Analyzer (DDA) [13] is an algorithm for drawing straight lines that sequentially finds the positions of the pixels to be drawn by adding the slope values of the lines. Mayacela et al. [14] constructed a discrete component emulator for memristors by implementing DDA in a Field Programmable Gate Array (FPGA) architecture.

Bresenham’s algorithm [15] is a fast DDA method for drawing straight lines. It can draw approximate line segments by sequentially adding successive points between a given starting point and an end point. Since this algorithm was designed for drawing line segments on a computer screen, it can be implemented using only integer additions, subtractions, and bit shifts, without using floating-point operations. Therefore, the cost of the computation is low and the processing speed can be increased. Murphy et al. [16] extended Bresenham’s algorithm and developed an algorithm for drawing thick line segments. Bhattacharya et al. [17] proposed a fast and scalable algorithm for estimating 2.5-dimensional shadow maps from Digital Surface Models (DSMs) derived from Light Detection And Ranging (LiDAR).

The problem with Bresenham’s algorithm is that if the line segment is not close enough to the center of the pixel, the pixel cannot be detected. Therefore, Bresenham’s algorithm cannot detect every pixel through which the line segment passes.

### 5.2. Ray-Tracing Method

In this paper, we use the ray-tracing method [12] to determine whether all pixels on a line segment are passable. Hu et al. [18] proposed a method for constructing a tomography model using observation data from ground-based Global Navigation Satellite System (GNSS) meteorological observation stations by applying a ray-tracing method to a fast voxel scanning algorithm.

**Figure 5:**
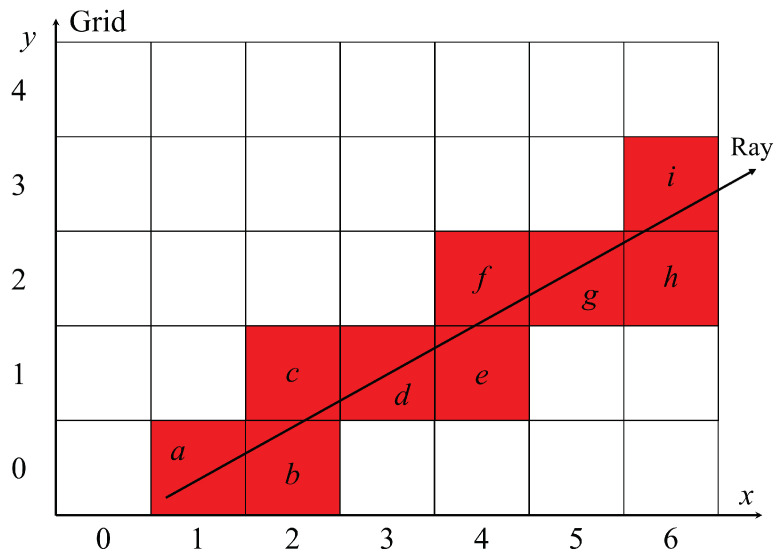
Order of pixels traversed by ray-tracing method.

Figure 5 illustrates a line segment between two points on a two-dimensional plane and the pixels that the line segment passes through using the ray-tracing method. In the figure, the ray-tracing method detects nine pixels in the two-dimensional plane through which the line segment starting at point (1, 0) passes, and these pixels are displayed in red.

The ray-tracing method detects all of the pixels through which the line segment that connects the start pixel to the end pixel passes. In order for the ray-tracing method to correctly determine the coordinates of all the pixels through which the line segment passes, it is necessary to detect pixels in the order a,b,c,d,e,f,g,h, and *i* through the half-line Ray.

In Figure 5, when a line segment moves from *a* to *b*, it crosses a pixel boundary with respect to the horizontal direction. When the segment moves from *b* to *c*, it crosses a pixel boundary in the vertical direction. Therefore, when Ray moves from the starting point and crosses a pixel boundary in the horizontal direction, the *x*-coordinate is incremented. When Ray crosses a vertical pixel boundary, the *y*-coordinate is incremented. In order to apply the ray-tracing method to three-dimensional space, we added a similar process for the *z*-axis to the two-dimensional ray-tracing method.

### 5.3. Procedure of Searching for Puncture Route

The proposed method detects the coordinates of voxels through which a line segment passes by applying a ray-tracing method in three-dimensional space. Based on the CT values of the detected voxels, the proposed method determines whether the line segment can be punctured.

**Figure 6:**
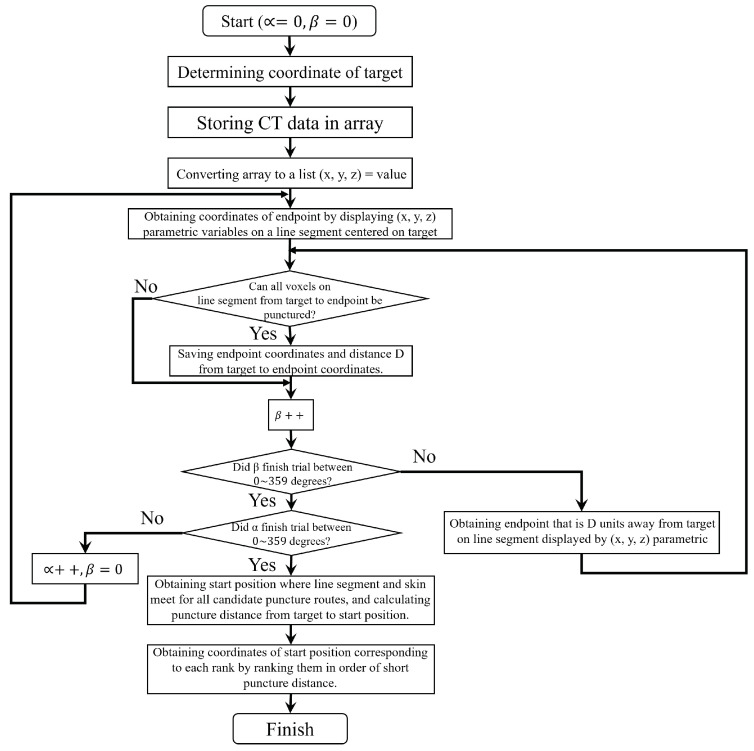
Flowchart of proposed method.

The flowchart of the proposed method is shown in Figure 6. The procedure used to find the puncture route in the proposed method is as follows:

1. The coordinates of the target to be punctured are determined based on the physician’s judgment;
2. The 4,439,040 (=512×512×170) items of CT data that compose the CT image including the target are stored in an array;
3. For each coordinate in three-dimensional space, a list of CT values stored in the array is created in the form (x,y,z)=value;
4. The endpoint coordinates are determined by drawing a line of sufficient length from the target coordinates to the exterior of the body. Based on the ray-tracing method, the coordinates of the voxel through which the line passes are obtained;
5. Based on the CT values of the coordinates obtained from the list created in step 3, the proposed method determines whether the line is puncturable. If so, the endpoint coordinates are stored;
6. To measure the distance from the target to the endpoint coordinates, Equation (1) is executed for 360 degrees, moving the target in 1 degree increments relative to the xy-plane. When the position of the endpoint coordinates is updated, steps 4 and 5 are performed for all 360-degree directions;
7. For the endpoint coordinates initially measured in step 4, Equation (1) is executed for 360 degrees, moving the coordinates along the *z*-axis by 1 degree. When the *z*-axis endpoint coordinates are updated, steps 4 through 6 are performed;
8. The coordinate of the contact point between the skin and the line that was determined to be puncture-ready in steps 5 through 7 is obtained. The contact point on the skin is represented by the coordinates of the voxel between the voxel with the CT value of the fat and the voxel with the CT value of the air part, which represents the exterior of the body. The distance from the coordinates of this voxel to the coordinates of the target is the puncture distance;
9. For multiple candidate routes for which the puncture distances have been measured in steps 5 to 8, the 360-degree search area is divided into multiple areas with a given puncture angle. The start coordinates of the puncture are derived in the order of the shortest puncture distance in each range.

### 5.4. Visualization of Puncture Route

**Figure 7:**
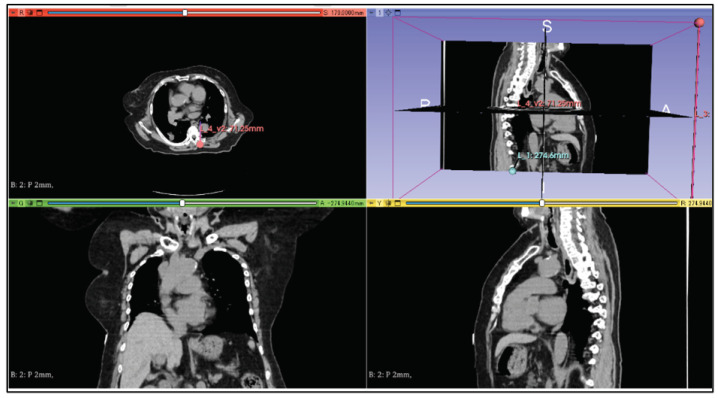
Example of a CT image using 3D Slicer.

By visualizing the line segment from the target to the coordinates of the puncture start position as a three-dimensional image, a physician can easily determine the puncture route. In this study, we used the 3D Slicer [19] application to visualize the puncture route. An example display of a CT image using the 3D Slicer is shown in Figure 7. 3D Slicer is open source software that can construct three-dimensional images from DICOM data.

**Figure 8:**
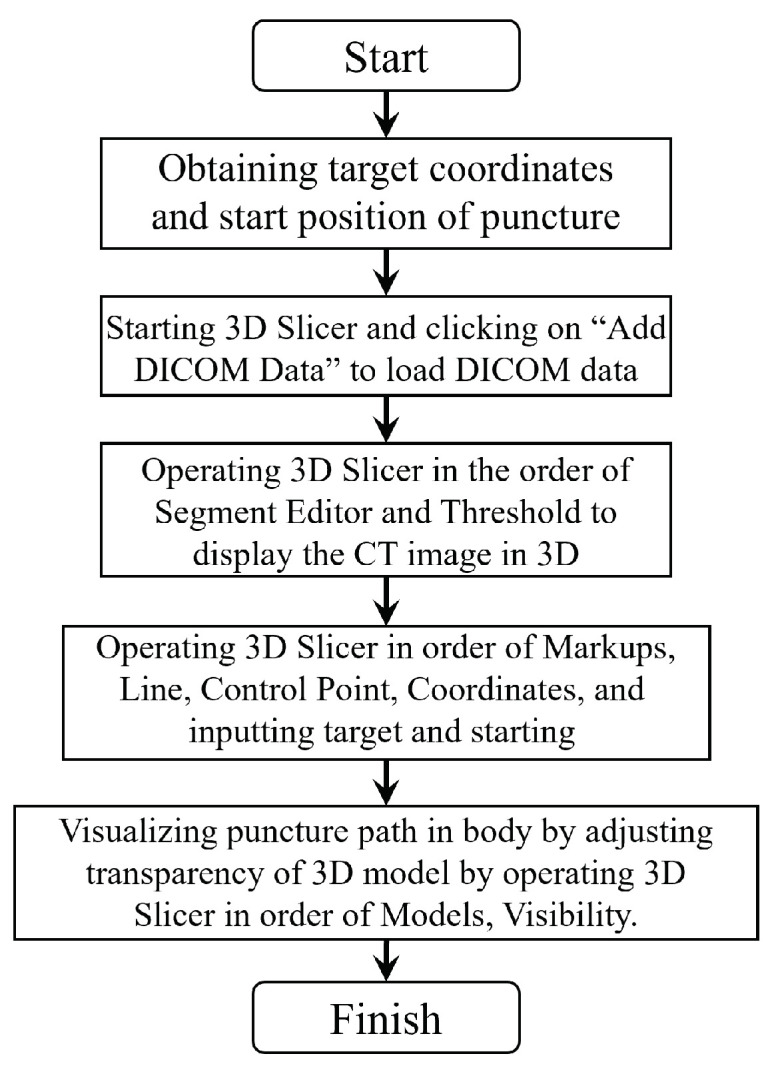
Flowchart of 3D Slicer.

The flowchart of 3D Slicer is shown in Figure 8. The procedure for visualizing the puncture route using 3D Slicer is as follows:

1. Based on the procedure for finding the puncture route using the proposed method described in Section 5.3, the coordinates of both the target and the starting coordinates of the puncture are obtained;
2. The “Add DICOM Data” button of 3D Slicer loads the DICOM data to be displayed with 3D Slicer;
3. Using 3D Slicer in the order of “Segment Editor” and “Threshold”, the CT image is displayed in 3D. In three-dimensional modeling, the range of CT values is adjusted according to the internal tissue;
4. After running 3D Slicer in the order of “Markups”, “Line”, “Control Point”, and “Coordinates”, the coordinates of the target and the start position of the puncture route to be visualized are loaded;
5. By operating 3D Slicer in the order of “Models” and “Visibility”, the transparency of the three-dimensional model is adjusted and the puncture route inside the body is visualized.

## 6. Evaluation

### 6.1. Evaluation Environment

We evaluated the usefulness of our proposed method for deriving the shortest route for CT-guided percutaneous punctures using CT image data. In the evaluation, we compared the processing time required to derive the puncture route between the proposed method and the brute-force method. We also evaluated the practicality of the proposed method based on a questionnaire regarding the puncture routes derived by the proposed method. The questionnaire was completed by physicians who actually perform puncture treatments.

The CT images are different for each patient, and the candidate puncture routes are also different. The proposed method focuses on deriving a three-dimensional puncture route within the allowed time in an actual puncture. In addition, in the questionnaire evaluation, several physicians indicated that the proposed method is useful based on the assumed environment.

**Figure 9:**
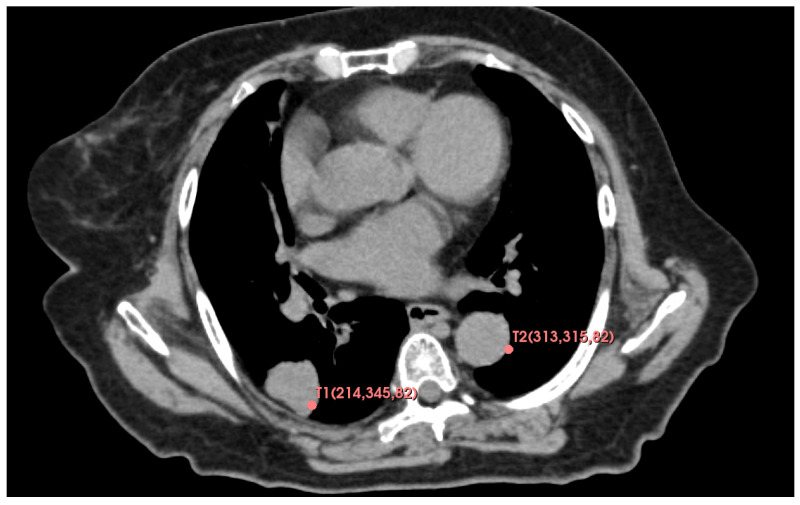
Coordinates of targets $T_{1}$ and $T_{2}$.

The performance of the computer used for the simulation evaluation is shown in Table 2. In this research, we used CT images of one patient with lung masses. In addition, we set two targets at $T_{1}$ and $T_{2}$ as shown in Figure 9. The coordinates of these two points were $T_{1}$= (214,345,82) and $T_{2}$= (313,315,82).

### 6.2. Processing Time to Derive Puncture

**Figure 10:**
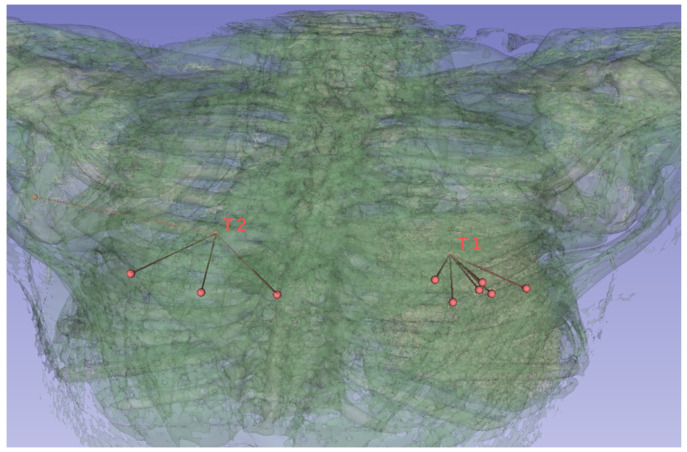
Example of three-dimensional puncture route.

Figure 10 shows the puncture routes derived by the proposed method. The red spheres in the figure are the start and end points of the puncture, and the line connecting the two red spheres is the puncture route. In this evaluation, the human body was composed of 170 two-dimensional CT images, and these images were superimposed to create a three-dimensional CT image. The processing time required to derive the puncture route for a three-dimensional CT image using all 170 two-dimensional CT images was approximately 59.4 s.

**Figure 11:**
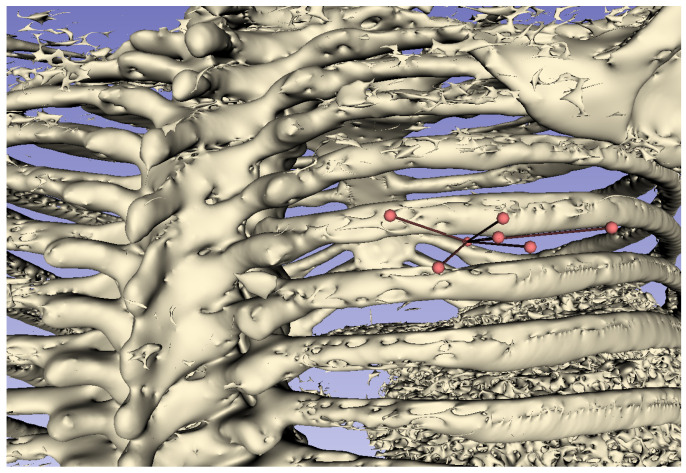
Visualization of puncture route for $T_{1}$.

**Figure 12:**
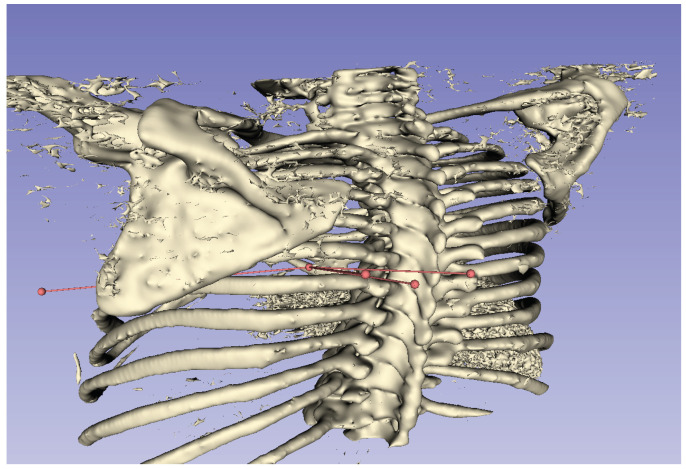
Visualization of puncture route for $T_{2}$.

For the puncture targets $T_{1}$ and $T_{2}$, the visualization of the puncture routes are shown in Figure 11 and Figure 12, respectively. These figures show that all puncture routes pass through the bone, indicating that they are not puncturable routes.

Next, the lengths of the puncture routes derived by the proposed method for $T_{1}$ and $T_{2}$ are shown in Table 3 and Table 4, respectively. These tables show that the coordinates of (X,Y,Z), which is the optimal starting position of the puncture to the target, are (205, 388, 80) for $T_{1}$ and (318, 275, 72) for $T_{2}$. The puncture distance is about 21.97 mm in $T_{1}$ and about 20.17 mm in $T_{2}$.

Table 3 shows that the length of the puncture route was almost the same for all ranks. On the other hand, the starting positions of ranks 9 and 10 in Table 4 were significantly different from those of ranks 1 to 8. The proposed method may derive a puncture route that is not assumed by the puncturing physician. Therefore, we showed that the proposed method can support the selection of the puncture route actually used by a puncture physician.

### 6.3. Comparison of Processing Times for Deriving Three-Dimensional Puncture Route

**Figure 13:**
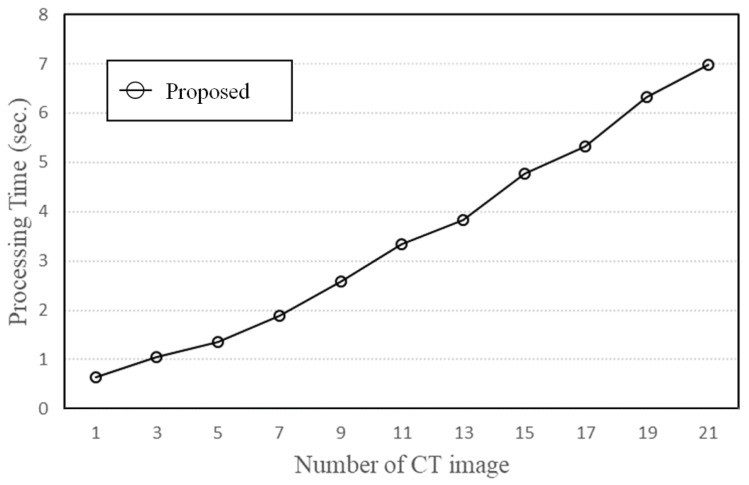
Processing time of proposed method.

**Figure 14:**
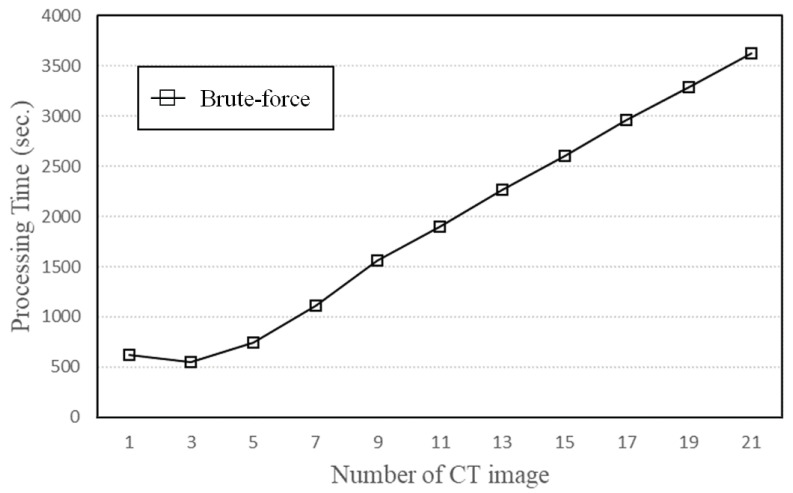
Processing time of brute-force method.

When the number of two-dimensional CT images constituting a three-dimensional CT image was changed, we calculated and compared the processing times to derive the puncture route for the proposed method and the brute-force method. The processing times of the proposed method and the brute-force method are shown in Figure 13 and Figure 14, respectively. The horizontal axis is the number of CT images, and the vertical axis is the processing time needed to search for the puncture route. As the number of CT images increases, the processing time becomes longer because the amount of data read increases and the search area expands.

In evaluations using a general-purpose computer, when the number of slices was 23 or more, the conventional method could not be evaluated due to application errors. Therefore, in this paper, we compared the processing times of the proposed method and the conventional method for slice numbers from 1 to 21.

We confirmed with physicians that it is possible to derive most of the puncture route candidates when the slice number is 15.

Figure 13 and Figure 14 show that the search time needed for 15 two-dimensional CT images was 4.77 s for the proposed method and 2599 s for the brute-force method. Therefore, the search time of the proposed method was about 99.8% shorter than that of the brute-force method. This significant reduction in processing time enables the proposed method to derive a larger number of candidate puncture routes for all human bodies without considering the number of CT images or the upper limit of search angles.

### 6.4. Usability Evaluation by Questionnaire

The usefulness of the puncture routes derived by the proposed method was evaluated by the puncture physicians using a questionnaire. There were three physicians, consisting of a man in his 40s (ID1), a woman in her 30s (ID2), and a man in his 30s (ID3). All of them had experience in puncture surgery. The evaluation was performed using a 5-point scale.

In a two-dimensional puncture, the puncture route can be derived from a single CT image containing the target. In this paper, we compared the proposed method and the brute-force method with a range of slice numbers from 1 to 21.

There are few physicians who perform punctures in medical settings. On the other hand, we proposed a method to derive a three-dimensional puncture route within the allowed time in actual punctures. In this paper, three physicians evaluated the operability of the proposed method from the perspective of practicality.

The evaluation results are shown in Table 5. The puncture physician responded that he was satisfied with the processing time and the puncture route using the proposed method. In addition, the physician also answered that the puncture routes indicated by the proposed method could be used in actual puncture procedures. On the other hand, the physician indicated that the operation of 3D Slicer for displaying the puncture route was complicated. Therefore, it is necessary to improve the method of displaying the puncture route.

## 7. Limitations and Potential

### 7.1. Improved Accuracy in Detecting Puncturable Areas

The proposed method determines the availability of a puncture route by referring to the CT values stored in voxels. However, the CT values of non-puncturable organs or tissues around organs may approximate the CT values of puncturable regions. In this case, the proposed method may preferentially derive a non-puncture route that is shorter than the candidate puncture routes.

In this paper, we set the range of CT values that can be punctured based on interviews with physicians. In normal organs, the shape is well formed and the CT values are uniform. On the other hand, in diseased organs, the shape changes and the CT values become non-uniform. The puncture route derived by the proposed method based on CT images containing diseased organs may include regions that cannot be punctured. Therefore, it is necessary to add various conditions, such as the locations of organs and the direction of the puncture from the target, in addition to the CT values, to determine whether a puncture route is actually available. In addition, the risk of error in three-dimensional puncture can be avoided by checking the CT image and the puncture route derived using the proposed method, and excluding from the list of candidates any puncture routes that include areas that cannot be punctured.

### 7.2. Expansion of Data Set

In order to improve the practicality of the proposed method, we needed to evaluate the data set using CT images of more patients. As a computational requirement, this paper shows that it is possible to derive a three-dimensional puncture route on a general computer. However, as the number of CT images used for evaluation increases, the data set becomes larger, and the search process becomes more complex. As a potential solution, the number of searches can be further reduced by clarifying the judgment of the puncture start position. In addition, the puncture area can be limited by setting the maximum length of the puncture needle. In this case, the number of CT images used is reduced, and the large-scale data set used in the proposed method can be suppressed.

### 7.3. Factors for Determining Selection Order of Puncture Routes

The proposed method ranks the many candidates in order of shortest puncture distance and presents them to the user as puncture routes. However, in an actual puncture, it is necessary to consider not only the shortest puncture distance but also a route that safely avoids areas that cannot be punctured, such as bones and internal organs. In addition, the preferred puncture route changes during the procedure depending on the location of the target and the patient’s physical condition. Therefore, the physician needs to be able to choose from a large number of candidates. A safe route can be derived by calculating the distance from each voxel constituting the puncture route to the nearest voxel in the non-puncturable region. Based on the ranks of the shortest route and the safe route, the proposed method can propose a route that is judged to be optimal.

### 7.4. Improving Operability by Visualizing Puncture Route

In the process of proposing a puncture route, the coordinates of the starting position of the puncture are derived, and these coordinates are loaded into 3D Slicer to be displayed on the screen. In the evaluation of the questionnaire, the puncture physician answered that the extraction of the coordinates by the proposed method and the visualization by 3D Slicer were cumbersome because they were separate operations. Since 3D Slicer is an open source software, we decided that it could be operated more intuitively by combining it with the coordinate detection program created by the proposed method.

### 7.5. Collaboration with Mixed Reality (MR) Technology

Puncture physicians have a heavy workload because they perform punctures to targets from outside the body while checking CT images. The proposed method reduces the processing time and workload of physicians by displaying the puncture route on an MR device and combining it with a puncture sensor device to check the status of the puncture while performing the procedure. In addition, regardless of the skill level of the physician, the needle can safely reach the target by following the puncture route derived by the proposed method. Therefore, the number of physicians who can perform puncture operations can be increased.

## 8. Conclusions

In this paper, we propose a method for finding a three-dimensional puncture route in CT-guided percutaneous punctures. The proposed method uses a ray-tracing method in three-dimensional space to quickly derive the pixel coordinates of the line segment passing from a given start point to an end point in a two-dimensional plane. This method can derive the puncture route from different angles without limiting the number of CT images used for the search or the angle of the puncture. In addition, the puncture routes derived by the ray-tracing method are visualized with 3D Slicer. The physician can, thus, visually assess the usefulness of the three-dimensional puncture route.

For a three-dimensional CT image created by combining 170 two-dimensional CT images, the processing time for deriving the puncture route using the proposed method was approximately 59.4 s. The shortest length of the puncture route from the starting point to the target was between 20 mm and 22 mm. In the evaluation of the processing time according to the number of CT images, we confirmed that the proposed method reduces the search time by approximately 99.8% compared to the brute-force method. In a questionnaire-based evaluation, physicians with experience in puncture treatment responded that the puncture route derived by the proposed method was useful.

Future plans include improving the recognition accuracy of the puncturable area and incorporating a factor that selects a puncture route with less burden on the patient.

## Figures and Tables

**Table 1:** CT values for types of tissue throughout the body.

Body Tissue	CT Values [HU]
Air in airway and gastrointestinal tract (specific gravity 0)	−2000∼−900
Adipose tissue	−100∼−50
Cerebrospinal fluid and ventricles	10
Periventricular white matter	20∼30
Cerebral cortex (gray matter)	30∼40
Vessels (and organs) (e.g., muscle and liver)	30∼60
Blood (specific gravity 1.05∼1.06)	50∼60
Coagulated blood (thrombus)	50∼100
Thyroid (specific gravity 1.10∼1.12)	100∼120
Bone and calcified lesions	250∼1000

**Table 2:** PC specifications.

CPU	Intel^®^Pentium(R) CPU G4400 (3.30 GHz) × 2
Memory	7.7 GBytes
OS	Ubuntu 18.04.1 LTS
Programming language	python 3.8.1
Supporting libraries	matplotlib 3.3.4
	numpy 1.20.1
	numpydoc 3.0.7
Memory usage	3.03 GBytes

**Table 3:** Puncture coordinates and puncture distances for $T_{1}$.

Rank	Puncture Coordinate	Puncture Distance (mm)
1	(205, 388, 80)	21.97
2	(210, 389, 82)	22.10
3	(204, 388, 78)	22.12
4	(209, 389, 85)	22.23
5	(200, 387, 75)	22.34
6	(205, 388, 72)	22.59
7	(212, 390, 88)	22.78
8	(216, 391, 72)	23.46
9	(192, 388, 82)	24.16
10	(228, 391, 88)	24.21

**Table 4:** Puncture coordinates and puncture distances for $T_{2}$.

Rank	Puncture Coordinate	Puncture Distance (mm)
1	(318, 275, 72)	20.17
2	(263, 299, 86)	26.37
3	(313, 386, 81)	35.50
4	(313, 386, 85)	35.56
5	(338, 387, 89)	38.32
6	(285, 389, 81)	39.56
7	(220, 280, 73)	49.84
8	(219, 278, 72)	50.71
9	(410, 245, 78)	59.83
10	(402, 230, 73)	61.66

**Table 5:** Questionnaire content.

Number	Question	Score		
		ID1	ID2	ID3
Q1	Is the display time of the candidate puncture routes in the proposed method within the acceptable range?	4	4	4
Q2	Could you actually use the candidate puncture routes derived by the proposed method?	4	3	3
Q3	Do you find the proposed puncture route acceptable?	5	3	3
Q4	Can you understand the operation until the route is displayed?	3	3	2
Q5	Do you really want to use the suggested method?	3	3	2

## Data Availability

The original contributions presented in the study are included in the article, further inquiries can be directed to the corresponding author.

## Abbreviations

The following abbreviations are used in this manuscript:

CT	Computed Tomography
CPU	Central Processing Unit
DICOM	Digital Imaging and Communications in Medicine
DDA	Digital Differential Analyzer
DSMs	Digital Surface Models
FPGA	Field Programmable Gate Array
GNSS	Global Navigation Satellite System
GPUs	Graphics Processing Units
HU	Hounsfield Unit
IVR	Interventional Radiology
LiDAR	Light Detection And Ranging
MR	Mixed Reality
OS	Operating System
US	Ultrasonography
